# Correction

**DOI:** 10.1080/15592324.2025.2542661

**Published:** 2025-08-20

**Article title**: Individual amino acid residues in CLV3 peptide contribute to its stability in vitro

**Authors**: Xiu-Fen Song, Ting-Ting Xu, Shi-Chao Ren and Chun-Ming Liu

**Journal**: *KPSB: Plant Signaling & Behavior*

**Bibliometrics**: Year 2013, Volume 8, Number 9, Article ID e25344

**DOI**: http://dx.doi.org/10.4161/psb.25344

We have identified an error in Figure 1P (highlighted in red in the original version) on page e25344-2. The figure is labeled as *clv3-2* treated with CLV3p12, but a SAM picture of L*er* wild type (WT) was misused. This mistake occurred due to the phenotype similarity between L*er* WT and *clv3-2* after treatment with CLV3p12, leading to the misuse of the picture. We are submitting a corrected version of Figure 1P (highlighted in red in the corrected version) that is a picture of *clv3-2* after treatment with CLV3p12. The text remains unchanged.

**Figure 1.**
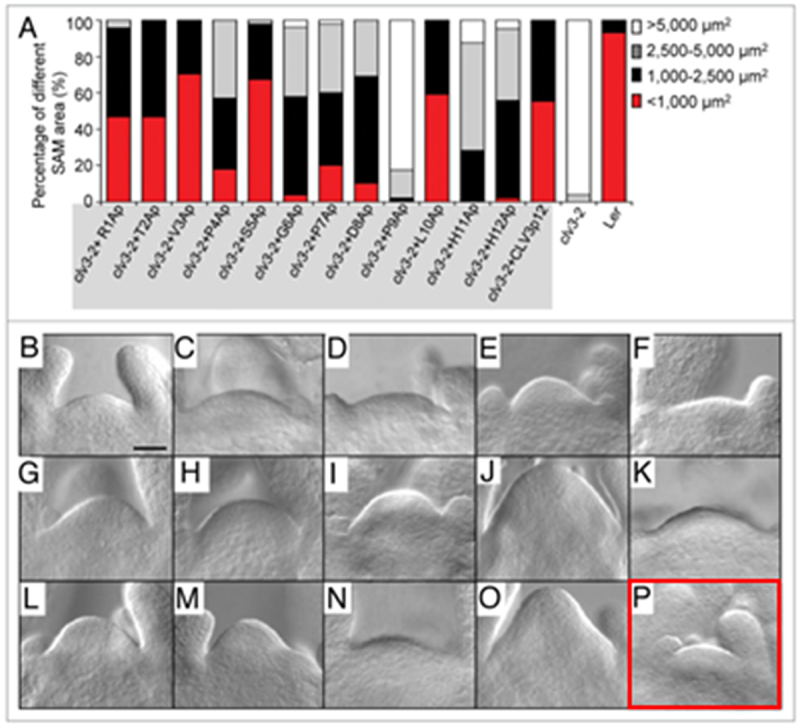
Efficiencies of Ala-substituted CLV3 peptides in restoring the enlarged SAM s of *clv3-2* in vitro. (**A**) Percentage of different SAM areas in *clv3-2* treated with Ala-substituted CLV3p12 peptides (shaded in gray) at the concentration of 10 μM. Red, < 1,000 μm^2^; black, 1,000–2500 μm^2^; gray, 2,500–5,000 μm2; white, > 5,000 μm^2^. (**B–P**) Representative pictures showing SAMs of *clv3-2* treated with Ala-substituted CLV3p12 peptides in vitro for 9 d. (**B**) R1Ap; (**C**) T2Ap; (**D**) V3Ap; (**E**) P4Ap; (**F**) S5Ap; (**G**) G6Ap; (**H**) P7Ap; (**I**) D8Ap; (**J**) P9Ap; (**K**) L10Ap; (**L**) H11Ap; (**M**) H12Ap. L*er* (**N**), *clv3-2* (**O**) and *clv3-2* treated with CLV3p12 (**P**) were used as controls. Bar in (**B**) = 50 μm for (**B–P**).

**Figure 1.**
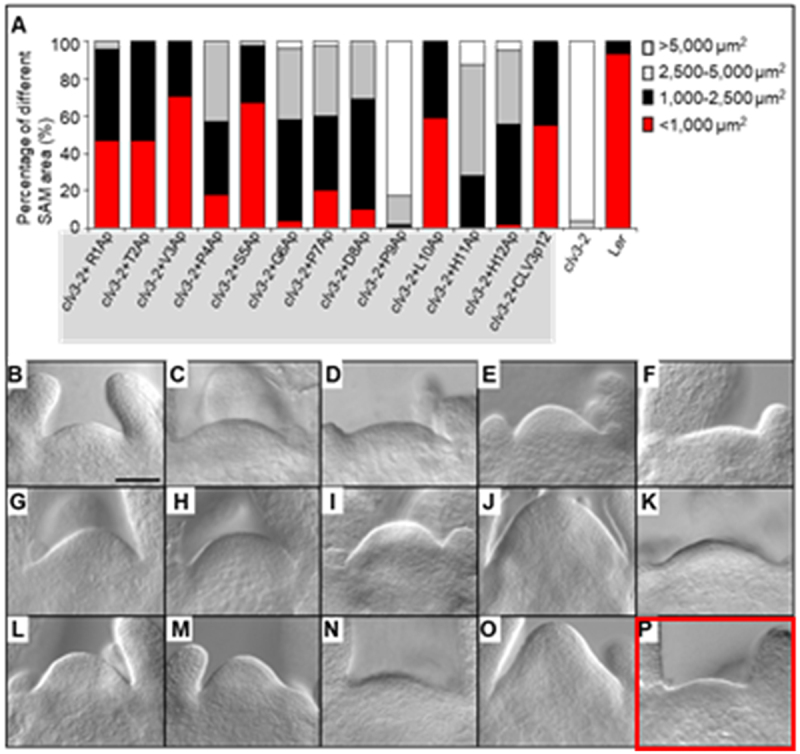
Efficiencies of Ala-substituted CLV3 peptides in restoring the enlarged SAM s of *clv3-2* in vitro. (**A**) Percentage of different SAM areas in *clv3-2* treated with Ala-substituted CLV3p12 peptides (shaded in gray) at the concentration of 10 μM. Red, < 1,000 μm^2^; black, 1,000–2500 μm^2^; gray, 2,500–5,000 μm2; white, > 5,000 μm^2^. (**B–P**) Representative pictures showing SAMs of *clv3-2* treated with Ala-substituted CLV3p12 peptides in vitro for 9 d. (**B**) R1Ap; (**C**) T2Ap; (**D**) V3Ap; (**E**) P4Ap; (**F**) S5Ap; (**G**) G6Ap; (**H**) P7Ap; (**I**) D8Ap; (**J**) P9Ap; (**K**) L10Ap; (**L**) H11Ap; (**M**) H12Ap. L*er* (**N**), *clv3-2* (**O**) and *clv3-2* treated with CLV3p12 (**P**) were used as controls. Bar in (**B**) = 50 μm for (**B–P**).

Attached below are the original and the corrected versions of Figure 1.

The original Figure 1, with the wrong photo P (framed in red, Song et al., 2013)

The corrected Figure 1, with the photo P replaced (framed in red, Song et al., 2013)

Reference:

Xiu-Fen Song, Ting-Ting Xu, Shi-Chao Ren and Chun-Ming Liu* (2013) Individual amino acid residues in CLV3 peptide contribute to its stability in vitro. Plant Signal. Behav., e25344.

